# Elevated monocyte-specific type I interferon signalling correlates positively with cardiac healing in myocardial infarct patients but interferon alpha application deteriorates myocardial healing in rats

**DOI:** 10.1007/s00395-018-0709-7

**Published:** 2018-11-12

**Authors:** Ellis N. ter Horst, Paul A. J. Krijnen, Nazanin Hakimzadeh, Lourens F. H. J. Robbers, Alexander Hirsch, Robin Nijveldt, Ingrid Lommerse, Ruud D. Fontijn, Elisa Meinster, Ronak Delewi, Niels van Royen, Felix Zijlstra, Albert C. van Rossum, C. Ellen van der Schoot, Tineke C. T. M. van der Pouw Kraan, Anton J. Horrevoets, Anja M. van der Laan, Hans W. M. Niessen, Jan J. Piek

**Affiliations:** 1 Department of Cardiology, Amsterdam UMC, University of Amsterdam, Meibergdreef 9, Amsterdam, The Netherlands; 2 Department of Biomedical Engineering and Physics, Amsterdam UMC, University of Amsterdam, Meibergdreef 9, Amsterdam, The Netherlands; 3 Netherlands Heart Institute, Moreelsepark 1, Utrecht, The Netherlands; 4 Department of Pathology, Amsterdam UMC, VU University Amsterdam, de Boelelaan 1117, 1081HV Amsterdam, The Netherlands; 5 Department of Cardiology, Amsterdam UMC, VU University Amsterdam, de Boelelaan 1117, Amsterdam, The Netherlands; 6 Department of Cardiac Surgery, Amsterdam UMC, VU University Amsterdam, de Boelelaan 1117, Amsterdam, The Netherlands; 7 Department of Molecular Cell Biology and Immunology, Amsterdam UMC, VU University Amsterdam, de Boelelaan 1117, Amsterdam, The Netherlands; 8 Amsterdam Cardiovascular Sciences, Amsterdam, The Netherlands; 9 Department of Experimental Immunohematology, Sanquin Research, Amsterdam UMC, location AMC, Plesmanlaan 125, Amsterdam, The Netherlands; 10 Department of Cardiology, Radboud University Medical Centre, Geert Grooteplein Zuid 10, Nijmegen, The Netherlands; 11 Department of Cardiology, Erasmus Medical Centre, Dr. Molewaterplein 40, Rotterdam, The Netherlands; 12 Department of Cardiology and Radiology, Erasmus Medical Centre, Dr. Molewaterplein 40, Rotterdam, The Netherlands

**Keywords:** Myocardial infarction, Interferon-α, Monocytes, Cardiac healing

## Abstract

Monocytes are involved in adverse left ventricular (LV) remodelling following myocardial infarction (MI). To provide therapeutic opportunities we aimed to identify gene transcripts in monocytes that relate to post-MI healing and evaluated intervention with the observed gene activity in a rat MI model. In 51 MI patients treated by primary percutaneous coronary intervention (PCI), the change in LV end-diastolic volume index (EDVi) from baseline to 4-month follow-up was assessed using cardiovascular magnetic resonance imaging (CMR). Circulating monocytes were collected at day 5 (Arterioscler Thromb Vasc Biol 35:1066–1070, 2015; Cell Stem Cell 16:477–487, 2015; Curr Med Chem 13:1877–1893, 2006) after primary PCI for transcriptome analysis. Transcriptional profiling and pathway analysis revealed that patients with a decreased LV EDVi showed an induction of type I interferon (IFN) signalling (type I IFN pathway: *P* value < 0.001; false discovery rate < 0.001). We subsequently administered 15,000 Units of IFN-α subcutaneously in a rat MI model for three consecutive days following MI. Cardiac function was measured using echocardiography and infarct size/cardiac inflammation using (immuno)-histochemical analysis. We found that IFN-α application deteriorated ventricular dilatation and increased infarct size at day 28 post-MI. Moreover, IFN-α changed the peripheral monocyte subset distribution towards the pro-inflammatory monocyte subset whereas in the myocardium, the presence of the alternative macrophage subset was increased at day 3 post-MI. Our findings suggest that induction of type I IFN signalling in human monocytes coincides with adverse LV remodelling. In rats, however, IFN-α administration deteriorated post-MI healing. These findings underscore important but also contradictory roles for the type I IFN response during cardiac healing following MI.

**Electronic supplementary material:**

The online version of this article (10.1007/s00395-018-0709-7) contains supplementary material, which is available to authorized users.

## Introduction

Adverse left ventricular (LV) remodelling is an important cause of heart failure and cardiac death after myocardial infarction (MI) [14]. Upon MI, an inflammatory response is initiated which involves recruitment of monocytes and macrophages into the infarcted site to promote removal of tissue debris and subsequently to stimulate tissue repair [22]. Monocytes consist of a heterogeneous pool of cells that are distinguished into specific subsets and can adapt their function in response to tissue injury [8, 22]. Although recruitment of monocytes is a prerequisite for proper infarct healing, excessive accumulation of activated monocyte and macrophage subsets into the myocardium may deleteriously affect post-infarct healing [22, 31]. Clinical studies have reported an association between the development of adverse LV remodelling and post-MI monocytosis [15, 18], indicating the importance of monocyte involvement in the pathogenesis of adverse LV remodelling. Hence, increased understanding of the monocyte response in MI patients is needed to provide new clues for treatments that could improve the LV remodelling process.

To beneficially influence the monocyte response following MI, several clinical studies have focused on unravelling specific monocyte surface markers to quantify and influence subset recruitment into the injured myocardium [5, 10, 19]. In an earlier study, we reported in MI patients that high levels of the peripheral pro-inflammatory monocyte subset were negatively associated with regional systolic function at 4-month follow-up [32]. However, functional gene transcripts of monocytes in MI patients that relate to adverse LV remodelling following MI have been poorly described, while this could provide essential therapeutic targets that influence monocyte functioning [16]. In the current study, we addressed this issue by investigating the transcriptome of circulating monocytes of the same MI patient group as our previous study [32] to subsequently correlate this with changes in cardiac function at 4-month follow-up. This revealed that enhanced signalling of the type I interferon (IFN) in monocytes of MI patients beneficially affects post-MI cardiac healing. These results implicate that systemic elevation of type I IFN may positively affect post-MI adverse LV remodelling. To evaluate this, we used a rat MI model and administrated the type I IFN, IFN-α, for three consecutive days following MI and analysed the effect of IFN-α on myocardial infarct size, cardiac function and the inflammatory response.

## Methods

Detailed methods are described in the supplementary material.

### Patient study and procedures

The present study is an ancillary study of the HEBE study, of which the study design, procedures and main results have been reported previously [9]. Briefly, the HEBE study was a multicentre, randomized trial, investigating the effect of intracoronary infusion of autologous bone marrow mononuclear cells and peripheral blood mononuclear cells (PBMC) after ST-segment elevation myocardial infarction. For this ancillary study, patients of the PBMC group were selected from which both the CMR and PBMC data were completely available (*n *=51). All patients underwent baseline CMR at 3 days [3, 4] after primary PCI, with a minimum of 2 days between PCI and CMR. With CMR, calculated functional parameters were indexed for body surface area as reported previously [9]. At 4-month follow-up, CMR was repeated and the change in LV end-diastolic volume index (EDVi) from baseline to 4-month follow-up was used as a measure of the extent of LV remodelling. Whole blood was collected at 5 days [4–6] after primary PCI. The study was conducted in accordance with the Declaration of Helsinki, and the study protocol was approved by Institutional Review Boards of the participating institutes. All patients gave informed consent. The trial was registered at the Netherlands Trial Register (#NTR166; www.trialregister.nl) and at the International Standard Randomized Controlled Trial register (#ISRCTN95796863; http://isrctn.org).

### Animals and experimental MI procedure

Male Wistar rats (*n* = 48, age 6–8 weeks, Harlan Laboratories, Horst, the Netherlands) weighing between 350 and 420 g were used. The study was approved by the VU University Amsterdam animal ethics and welfare committee. The VU University Amsterdam is licensed according to the 2010/63/EU guidelines. The rats involved in the current study were accommodated and cared for according to the guidelines described in appendix A of EST No. 123. Induction of MI and the use of medetomidine–sufentanil as anaesthesia are performed as described previously [29]. Briefly, the left anterior descending artery (LAD) was ligated for 40 min, followed by reperfusion and closure of the thorax. Post-MI analgesia was continued as suggested earlier [29]. Sham animals underwent the exact same procedures except for ligation of the LAD.

### Experimental setup

Following MI or sham, rats received 15,000 Units of mammalian rat Interferon Alpha (PBL Assay science) subcutaneously, diluted in 1 mL saline within 30 min following the surgical procedure for three consecutive days. The doses of subcutaneous 15,000 Units/mL was based upon pharmacokinetic properties of type I IFN [17], and usage of type I IFN in (pre)clinical settings during inflammatory diseases [21, 28, 34]. A subgroup was killed at 3 days following MI or sham (day 3) (MI: *n* = 10, sham: *n* = 3) and the remainder at 28 days following MI or sham (day 28) (MI: *n* = 8, sham: *n* = 3). Placebo MI and sham rats were treated equally but received a placebo subcutaneous injection containing 1 mL saline (day 3 MI *n* = 8, sham *n* = 4; day 28 MI *n* = 8, sham *n* = 4).

### Statistical analysis

Data with normal distribution are expressed as mean ± SD and data with non-normal distribution are given as median value (25th–75th percentile). Categorical data are presented as number (%). To test for differences between groups with a normal distribution, the Student’s *t* test was used and for data with a non-normal distribution, the Mann–Whitney *U* test was used for unpaired data and the Wilcoxon signed rank test for paired data. The Fisher’s exact test or the Chi-square test was used for testing associations between categorical data. A two-sided *P* value < 0.05 was considered statistically significant. Statistical analysis was performed with Statistical Package for Social Sciences software (SPSS 22.0 for Windows, SPSS Inc.).

## Results

### Patient characteristics

**Fig. 1:**
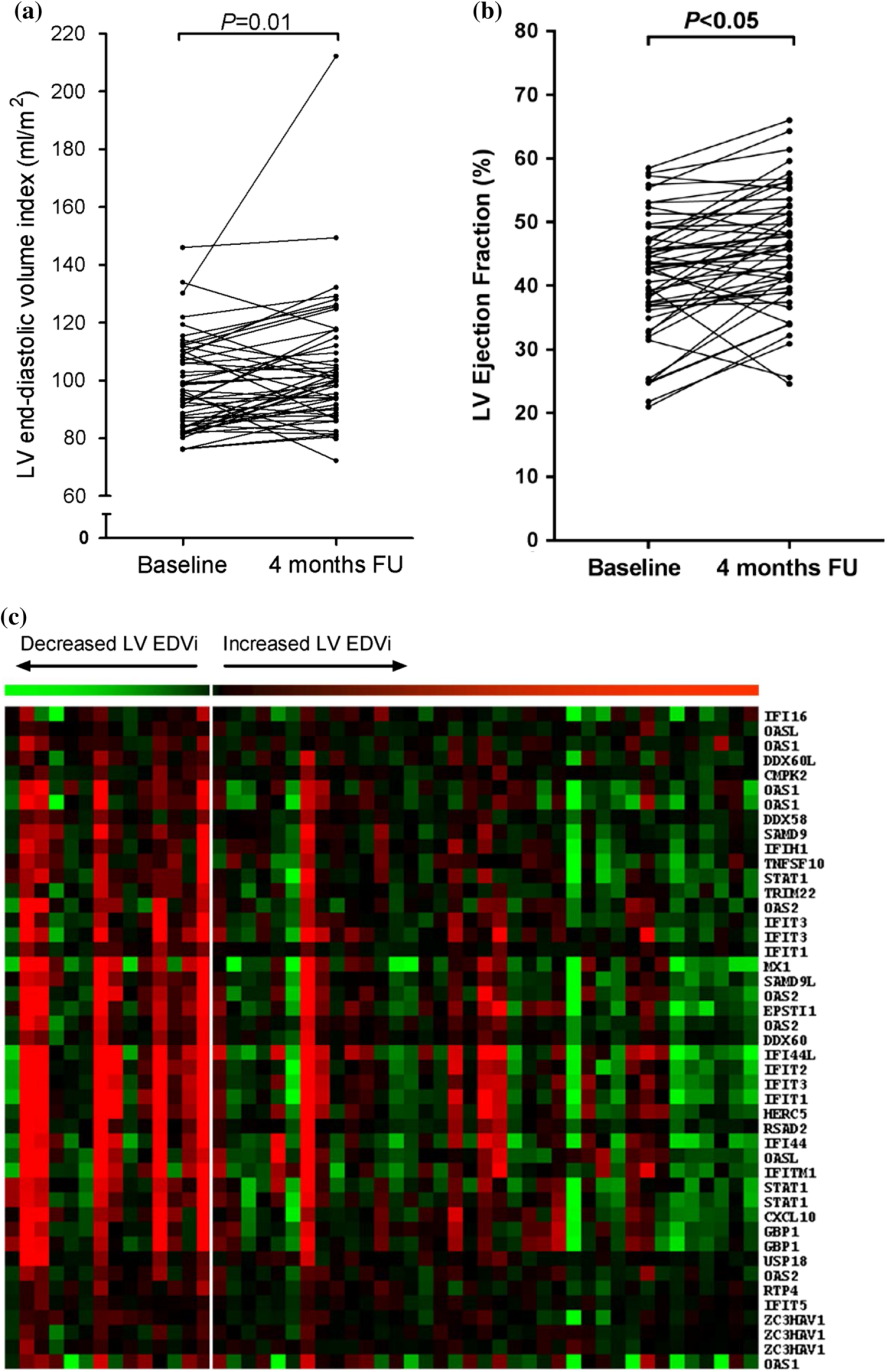
Increased IFN type I expression in monocytes of MI patients with a decreased EDVi baseline–4-month FU. **a** The change in LV EDVi from baseline to 4-month follow-up. **b** The change in LV EF from baseline to 4-month follow-up. **c** Heat map showing the expression of genes of the MOSERLE_IFNA_RESPONSE pathway supervised by the change in LV EDVi from baseline to 4-month follow-up. Each column represents one patient. Patients with a decreased LV EDVi at 4-month follow-up are placed on the left side (*n* = 14) and patients with an increased LV EDVi on the right side (*n* = 37). Red denotes relatively high gene expression, green represents relatively low-expressed genes. Note the relative high expression of genes of the MOSERLE_IFNA_RESPONSE pathway in patients with a decreased LV EDVi at 4-month post-MI (right side). Statistical analyses are performed using the Student’s *t* test. *EDVi* end-diastolic volume index, *EF* ejection fraction, *FU* follow-up, *LV* left ventricular

The baseline characteristics of the patient study population are shown in Supplementary Table S2. The mean age of the study population was 56 ± 9 years, 82% was male, and the median time from onset of symptoms to reperfusion therapy was 3.0 (2.2–4.8) h. Mean baseline infarct size was 18 ± 9% of the LV. A marked variety was observed in the change of LV EDVi from baseline to 4-month follow-up amongst patients. The LV EDVi increased from 98 ± 16 mL/m^2^ at baseline to 104 ± 22 mL/m^2^ at follow-up (*P* = 0.01), with a change in LV EDVi of 6 ± 15% (Fig. 1a). At 4-month follow-up, 37 patients (73%) showed an increase in LV EDVi. Supplementary Table S2 shows the baseline characteristics of the study population after stratification by change in LV EDVi. Mean LV ejection fraction (EF) was 42 ± 10% at baseline and was increased to 46 ± 9% at 4-month follow-up (*P *<0.05), with a change in LV EF of 4 ± 7% (Fig. 1b).

### Patients without adverse LV remodelling show induced type I IFN signalling

To identify gene transcripts that are significantly related to adverse LV remodelling following MI, we compared the transcriptome of monocytes between patients with a decreased LV EDVi and patients with an increased LV EDVi at 4-month follow-up. Whole genome transcriptome analysis revealed a total of 47 probes (45 unique genes) that were significantly differentially expressed (Supplementary Table S3). All significant genes were expressed at higher levels in patients with a decreased LV EDVi. Amongst these were several IFN-stimulated genes (ISGs). Verification of gene array expression data by real-time RT-PCR was performed for a selection of ISGs and agreed with the gene array data (Supplementary Table S4). Next, we performed transcriptome analysis at the pathway level. The top ten significant pathways were all expressed at higher levels in patients with a decreased LV EDVi, and were all related to IFN signalling (Table 1). The most significant pathway was the MOSERLE_IFNA_RESPONSE pathway (*P* value < 0.001; false discovery rate < 0.001), containing 50 genes that are upregulated in ovarian cancer progenitor cells in response to the type I IFN, IFN-α [20]. Figure 1c shows the expression of this type I IFN signalling pathway in relation to the change in LV EDVi, visualized in a heatmap. Furthermore, analysis of transcription factor-binding sites of genes that were expressed at significantly higher levels in patients with a decreased LV EDVi revealed transcription factor-binding motifs involved in IFN signalling, corroborating the above findings (Supplementary Table S5).

**Table 1:** Pathway analysis

Gene set name	Score	*P* value	FDR
MOSERLE_IFNA_RESPONSE	− 3.33	0.000	0.000
UROSEVIC_RESPONSE_TO_IMIQUIMOD	− 2.83	0.000	0.000
ZHANG_INTERFERON_RESPONSE	− 2.74	0.000	0.000
EINAV_INTERFERON_SIGNATURE_IN_CANCER	− 2.65	0.000	0.000
BENNETT_SYSTEMIC_LUPUS_ERYTHEMATOSUS	− 2.5	0.000	0.000
DAUER_STAT3_TARGETS_DN	− 2.37	0.000	0.000
BROWNE_INTERFERON_RESPONSIVE_GENES	− 2.28	0.000	0.000
RADAEVA_RESPONSE_TO_IFNA1_UP	− 1.87	0.000	0.000
ZHU_CMV_8_HR_UP	− 1.74	0.000	0.000
SANA_RESPONSE_TO_IFNG_UP	− 1.72	0.000	0.000

The change in LV EDVi was used as a quantitative parameter to identify significantly associated pathways. The top ten significant pathways are listed (sorted by score). Patients with an increased LV EDVi at 4-month follow-up showed attenuated type I IFN signalling

*FDR* false discovery rate

### Administration of IFN-α following experimental MI results in LV dilatation

Since elevated type I IFN signalling in monocytes of MI patients coincided with reduced adverse LV remodelling, we hypothesized that systemic elevation through type I IFN protein could exert a comparable effect. To assess this, we used a rat MI model and systemically administered IFN-α following MI for three consecutive days. Rats that received a placebo injection served as control. The effect of MI on the endogenous expression of ISGs in rat PBMC and the effect of IFN-α administration hereon were primarily evaluated. This showed that MI caused increased *Cxcl10* mRNA expression levels from day 2 to day 3 and IFN-α administration increased its expression at day 2 (Supplementary Figure S1). This suggests that exogenous IFN-α administration could induce an early expression of ISGs in circulating PBMC following MI.

**Fig. 2:**
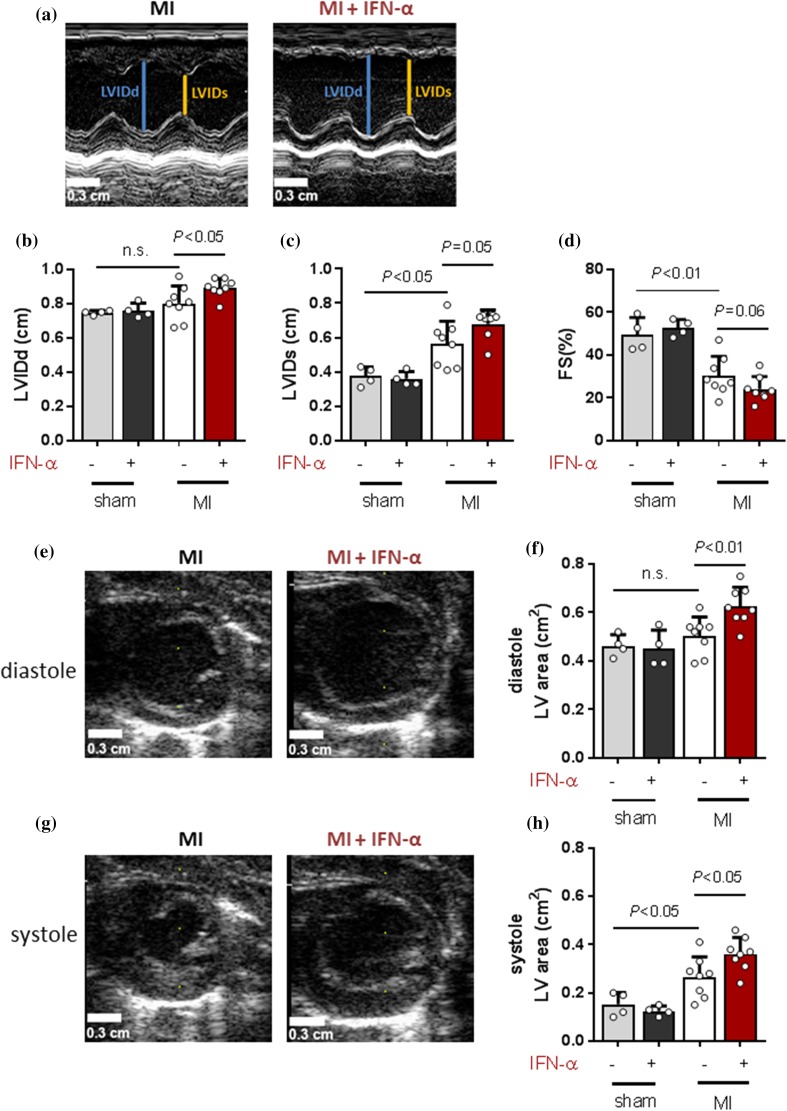
IFN-α administration results in ventricular dilatation in rats. 2D echocardiography measurements at day 28 showing **a** images of the M-mode parameters LVIDd and LVIDs which are quantified and presented in **b** and **c** showing mean ± SD. **d** Relative change in LVIDd and LVIDs presented as FS(%). Short axis of the LV area at day 28 in diastole in **e** (image) and **f** (quantification) as well as in systole in **g** (image) and **h** (quantification). *MI* myocardial infarction, *IFN-α* interferon alpha, *LVIDd* left ventricle internal diameter in diastole and *LVIDs* left ventricle internal diameter in systole. Statistical analysis are performed using the Student’s *t* test

To determine the effect of IFN-α application on post-MI adverse LV remodelling, echocardiography was performed (Fig. 2a, e and g). This showed that MI caused ventricular dilatation, shown by a significant increase of the systolic left ventricle internal diameter (LVIDs) (Fig. 2c; *P* < 0.05) and area (Fig. 2h; *P* < 0.05) in comparison to sham at day 28. Post-MI IFN-α administration deteriorated ventricular dilatation at day 28, shown by a further increase of LVID in diastole (Fig. 2b; *P* < 0.05) and of the LV area at day 28 in both diastole (Fig. 2f; *P* < 0.01) and systole (Fig. 2h; *P* < 0.05). Also, we showed that MI decreased fractional shortening (FS), reflecting a reduction in cardiac functioning. Post-MI IFN-α also decreased FS, although not significantly different in comparison to placebo (Fig. 2d; *P* = 0.06). In sham rats, IFN-α administration did not affect the measured cardiac parameters (Fig. 2b–d, f and h).

### IFN-α administration increases infarct necrosis and infarct size

**Fig. 3:**
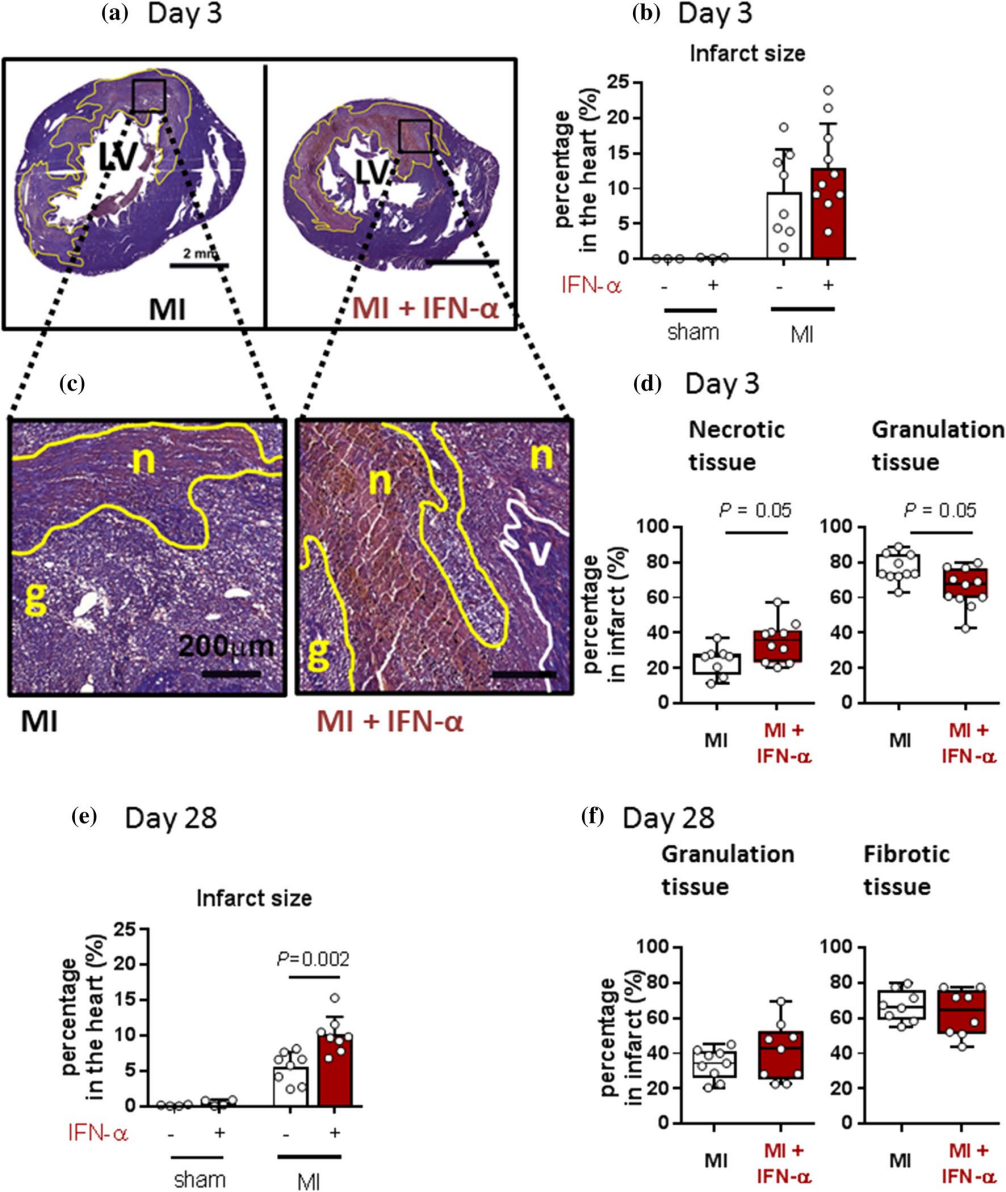
IFN-α administration increases the necrotic tissue area at day 3 and the total infarct size at day 28. **a** Images of a rat myocardial PTAH stain at day 3 with the infarcted area indicated by a yellow line. Scale bar represents 2 mm. **b** Quantification of the infarcted areas at day 3. **c** Magnification of the PTAH stain showing the infarcted area with necrotic (*n*) and granulation tissue (*g*) indicated by yellow lines and the viable myocardium (*v*) indicated by white lines. Scale bar represents 200 µm. **d** Quantification of necrotic and granulation tissue in the infarcted area at day 3. *P* values were calculated using the Mann–Whitney *U* test. **e** Quantification of the infarcted area at day 28 and **f** its quantification of granulation and fibrotic tissue. *PTAH* phosphotungstic acid–hematoxylin, *MI* myocardial infarction, *IFN-α* interferon alpha. Statistical analyses are performed using the Student’s *t* test (**b** and **e**) or the Mann–Whitney *U* test (**d** and **f**)

To determine the effect of IFN-α on post-MI cardiac healing, we evaluated the infarct size and the percentage of necrotic and granulation tissue at day 3 and the percentage of granulation and fibrotic tissue at day 28 via histochemical analysis (Fig. 3). In sham animals, no damaged myocardial tissue was found, nor after IFN-α administration (Fig. 3b). In MI rats, IFN-α administration resulted in more necrotic tissue and less granulation tissue at day 3 as compared to placebo (Fig. 3c, d; *P* = 0.05) whereas at day 28, no difference in infarct tissue composition was detected (Fig. 3f). However, post-MI IFN-α administration resulted in a significantly larger infarct area at day 28 in comparison to placebo (Fig. 3e; *P* < 0.01).

### Systemic IFN-α administration following MI affects monocyte and macrophage subset distribution

**Fig. 4:**
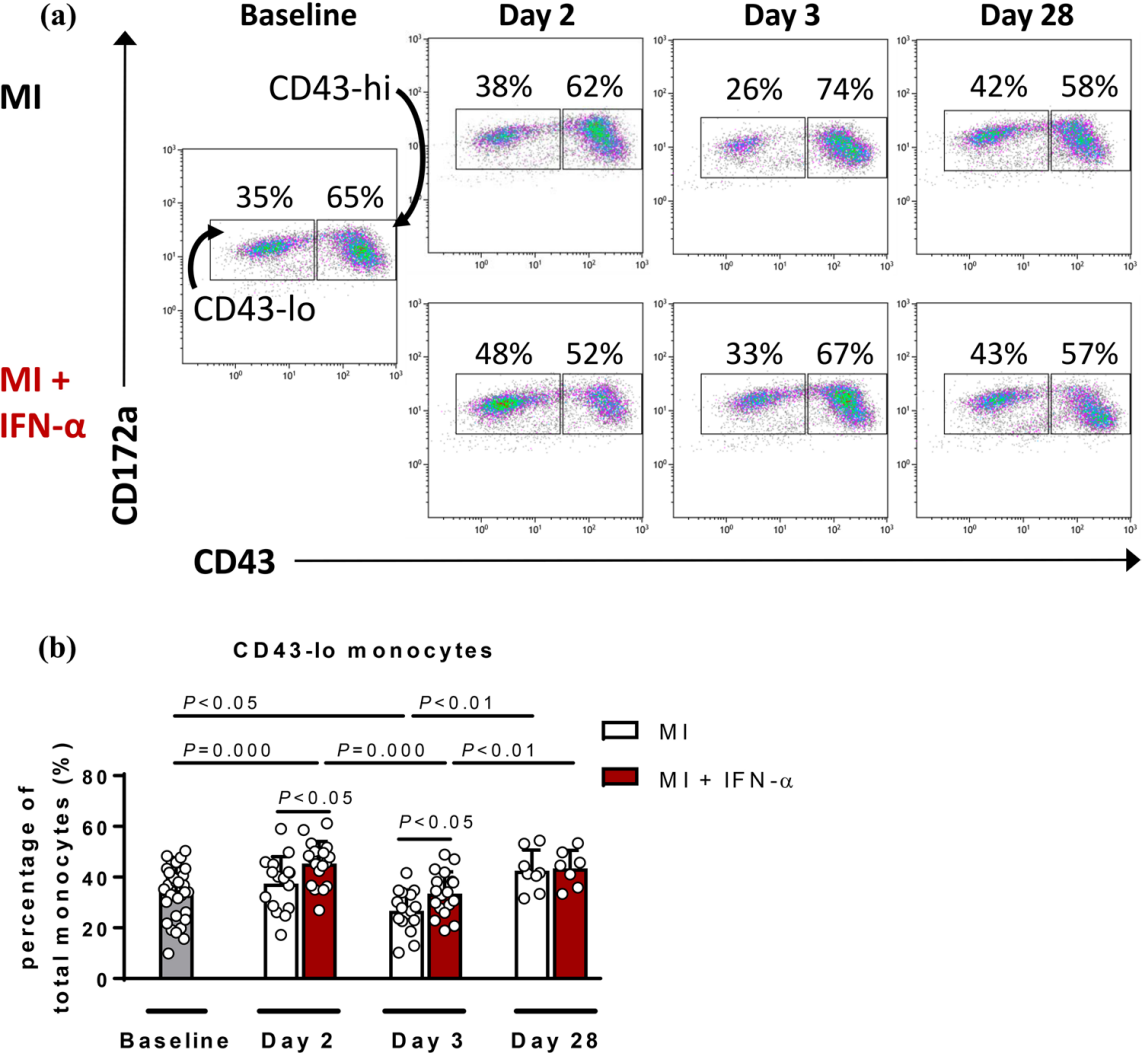
IFN-α changes the distribution of circulating monocyte subsets at day 2 following experimental MI. **a** Representative flow cytometry plots of mononuclear cells at baseline, days 2, 3 and 28. After baseline, rats were subdivided into groups (MI or MI + IFN-α). **b** Quantification of the pro-inflammatory CD43-lo monocytes in MI and in IFN-α-treated MI rats (red bars) at the different time points. *MI* myocardial infarction, *IFN-α* interferon alpha. Statistical analyses are performed using the Student’s *t* test (paired and unpaired)

As an exaggerated monocyte response has been shown to highly correlate with increased infarct size and deteriorated cardiac function [31, 32] and IFN-α has been shown to modulate immune responses [7], we analysed the effect of IFN-α administration on circulating monocyte subset distribution and the level of macrophages within the infarcted area. Using flow cytometry analysis, circulating rat monocytes were subdivided into pro-inflammatory classical subsets (CD43-lo) and reparative non-classical subsets (CD43-hi; Fig. 4a). This showed that following MI, the percentage of CD43-lo monocytes is significantly reduced at day 3 in comparison to baseline (*P* < 0.05) (Fig. 4b). Post-MI IFN-α administration significantly increased CD43-lo monocytes already at day 2 in comparison to baseline (*P* = 0.000) (Fig. 4b, red bars) and also induced a significant increase of the CD43-lo monocytes at both day 2 (*P* < 0.05) and day 3 (*P* < 0.05) in comparison to placebo (Fig. 4b).

**Fig. 5:**
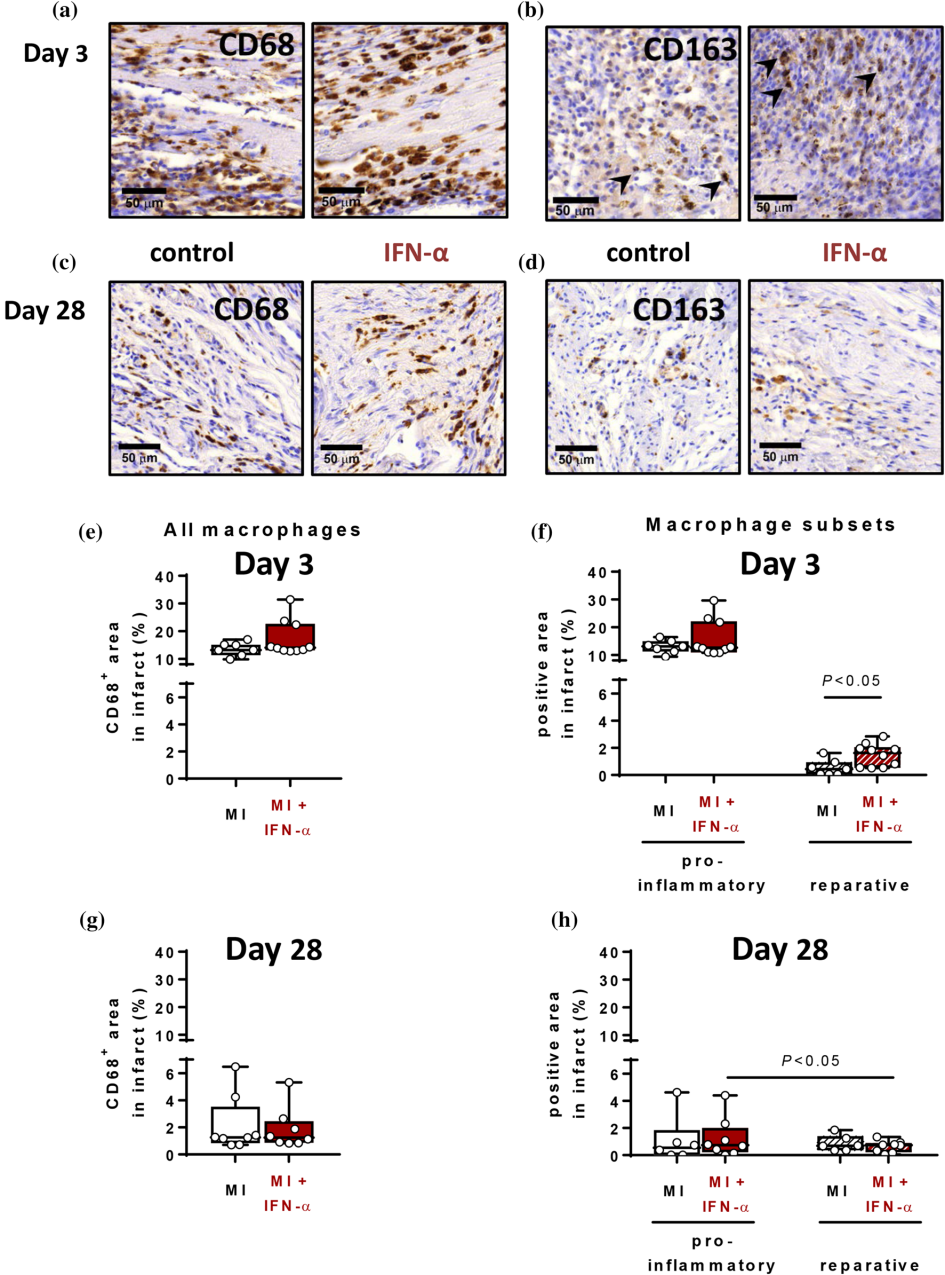
IFN-α does not alter total macrophage infiltration in the myocardium but increases alternative macrophage infiltration into the myocardium at day 3. Immunohistochemical images of the infarcted myocardium at day 3 (**a**–**b**) and day 28 (**c**–**d**) of CD68 (**a** and **c**) staining of all macrophages and CD163 (**b** and **d**) representing the reparative alternative macrophages. Scale bar represents 50 µm. Quantification of CD68 at day 3 (**e**) and day 28 (**g**) and the quantification of the macrophage subsets at day 3 (**f**) and day 28 (**h**). *MI* myocardial infarction, *IFN-α* interferon alpha. Data are presented as box plots showing the median and 25th–75th percentile together with the minimum and the maximum values. Statistical analyses are performed using the Mann–Whitney *U* test (unpaired) or the Wilcoxon signed rank test (paired)

Using quantitative immunohistochemical analysis (Fig. 5a–c), we showed that IFN-α administration did not affect the total CD68^+^ macrophages infiltrate at day 3 (Fig. 5e) or at day 28 (Fig. 5g). However, at day 3, post-MI IFN-α administration significantly elevated the alternative macrophages, without affecting the pro-inflammatory macrophage abundance (Fig. 5f; *P* < 0.05). Moreover, at day 28, MI showed an equal distribution of pro-inflammatory classical and reparative alternative macrophages in the infarct. IFN-α administration, however, resulted in a distribution towards more classical macrophages than alternative macrophages in the infarcted myocardium (Fig. 5h; *P* < 0.05), albeit this was not significantly different in comparison to placebo.

## Discussion

In the current study, we show contradictory roles of type I IFN and its signalling pathway in post-MI cardiac healing. In patients, transcriptome analysis in circulating monocytes of MI patients revealed that an induced expression of ISGs was associated with a decreased and, therefore, more preserved LV EDVi at 4-month follow-up. This profile represented the type I IFN signalling pathway suggesting that type I IFNs, and hence induction of type I IFN signalling in monocytes, positively correlate with beneficial post-MI LV remodelling in patients. However, in an experimental rat MI model, we showed that administration of exogenous IFN-α protein following MI resulted in more necrotic and less granulation tissue at day 3 and also exaggerated left ventricular dilatation together with an increased infarct size at day 28 in comparison to placebo-treated MI. Moreover, IFN-α administration increased the monocyte subset distribution following MI towards the more inflammatory CD43-lo subset at day 2 and day 3 whereas in the myocardium, the alternative macrophage subset was more present at day 3 after IFN-α administration. These results indicate that in a rat MI model, IFN-α application deteriorated the cardiac healing process following MI.

In patients, there is a marked difference in outcome following MI. Adverse LV remodelling occurs in approximately 15–50% of MI patients and is associated with heart failure and cardiac death [14, 35]. Monocytes have been implicated in the pathophysiology of adverse LV remodelling post-MI [4, 5] and with the current study, we are the first to investigate the gene transcripts of circulating monocytes in relation to the LV remodelling process following MI.

Sufficient healing of the infarcted area has been linked by several studies to a well-balanced inflammatory response. [6, 12, 22, 33] Type I IFN activation can either induce inflammation following a viral or bacterial infection, or contain a suppressive role in certain chronic infections [1, 11]. Variability in this response is highly dependent on cell type, pathogens and the environmental context [1]. In rats, we showed that post-MI IFN-α administration altered the distribution of circulating monocyte subsets towards the pro-inflammatory CD43-lo monocyte at day 2 in comparison to placebo, a shift which has generally been associated with an expanded infarcted area [31, 32]. Interestingly, in the infarcted area of the myocardium, post-MI IFN-α administration increased the presence of the reparative alternative macrophage subset at day 3. Even though pro-inflammatory macrophages were still predominantly present in the infarct area at that time point, this could implicate that IFN-α initiates an early propagation towards suppressing inflammation in the infarct in comparison to placebo MI rats [24, 30]. Remarkably, this also reveals that circulatory inflammatory cells do not always directly reflect the inflammatory status of the injured tissue. It has been suggested that type I IFN can affect the recruitment of monocytes and other inflammatory cells into the infarcted area, which could account for this observed difference between circulating and tissue-resident monocytes [3, 13, 34]. However, we did not find an effect of IFN-α on the number of macrophages in the myocardium early after MI, suggesting that IFN-α could have affected the inflammatory response through other mechanisms. Additionally, we showed that post-MI IFN-α administration increased the infarcted area at day 28 and deteriorated ventricular dilatation, an important predictor of heart failure and ventricular arrhythmia development [27]. Moreover, IFN-α administration resulted in an increase of necrotic tissue and a decrease in granulation tissue at day 3 in comparison to placebo MI. This could implicate that administration of IFN-α delayed the infarct healing, ensuing from a lack of cell debris clearance and replacement with granulation tissue.

Recently, King et al. published a study in which they demonstrate a negative effect of type I IFN signalling during healing following permanent MI in mice [13]. Type I IFN activation occurs classically through the IFN-α receptor (IFNAR). This induces activation of the Jak–Stat pathway resulting in Stat1 and Stat2 dimerization that initiates nuclear translocation of IFN regulatory transcription factors (IRF) and subsequent transcription of ISGs [1, 7, 23]. King et al. demonstrated that MI activates IRF3-dependent signalling through macrophages upon dsDNA sensing at day 4 following MI. Induction of MI in *irf3*^−*/*−^ mice showed reduced ventricular dilatation together with a marked decrease in infiltrated pro-inflammatory monocytes in comparison to MI in wild-type (wt) mice [13]. Moreover, King et al. showed that treatment with an IFNΑR-neutralizing antibody at 12 and 48 h after coronary artery ligation in wt mice improved cardiac functioning and survival in comparison to untreated wt mice following MI [13]. They concluded that systemic absence of endogenous type I IFN signalling, even when initiated after coronary ligation, beneficially affects cardiac healing following permanent MI. This supports our current results in rats showing that exogenous IFN-α administration following MI deteriorated cardiac healing.

Exaggerated IFN responses are increasingly associated with human autoimmune diseases and could detrimentally affect the functioning of type I IFN [1]. Also, the concentration and duration of IFN production together with specific timing of action can be crucial for accurate IFN functioning [1, 2]. In this regard, the findings that systemic IFN-α administration in rats and absence of type I IFN signalling in mice exerted a contradictory response in comparison to MI patients could implicate that elevated type I IFN signalling in monocytes does not reflect systemic elevated type I IFN signalling. Also, we can currently not exclude the response and contribution of non-myeloid cells after systemic IFN-α administration in the rats which could contribute to a different response in comparison to monocyte-specific type I IFN elevation in patients after MI. Remarkably, following arterial ischaemia, both increased type I IFN-related gene expression and type I IFN protein levels have been shown to negatively influence angio- and arteriogenesis in mice and human [25, 26]. Conceivably, timely activation of type I IFN signalling to promote tissue repair requires precise modulation through different immune cells.

In conclusion, we showed that in patients, monocyte-specific upregulation of ISGs involved in type I IFN signalling coincides with beneficial cardiac healing post-MI, whereas systemic IFN-α administration in a rat MI model detrimentally affected post-MI cardiac healing. These findings underscore the importance of the type I IFN response in cardiac healing, although exact regulatory mechanisms require further investigations.

## Electronic supplementary material

Below is the link to the electronic supplementary material.

Supplementary material 1 (DOCX 274 kb)
