# Supplementary material for: Elevated monocyte-specific type I interferon signalling correlates positively with cardiac healing in myocardial infarct patients but interferon alpha application deteriorates myocardial healing in rats
Source: Basic Res Cardiol. 2018 Nov 12;114(1):1. doi: 10.1007/s00395-018-0709-7 (PMC6244641; doi:10.1007/s00395-018-0709-7)
Supplement: Supplementary file 1 — Supplementary material 1 (DOCX 274 kb) [file 395_2018_709_MOESM1_ESM.docx]

Supplementary material

**Elevated monocyte specific type I interferon signalling correlates positively with cardiac healing in myocardial infarct patients but interferon-alpha application deteriorates myocardial healing in rats**

Ellis N. ter Horst PhD ^1,3,4,8*^, Paul A.J. Krijnen PhD^4,8^, Nazanin Hakimzadeh PhD^1,2^, Lourens F.H.J. Robbers MD, PhD^5^, Alexander Hirsch MD, PhD^12^, Robin Nijveldt MD, PhD^5^, Ingrid Lommerse^9^, Ruud D. Fontijn PhD^7^, Elisa Meinster^4,7^ Ronak Delewi MD, PhD^1^, Niels van Royen MD, PhD^10^, Felix Zijlstra MD, PhD^11^, Albert C. van Rossum MD, PhD^5^, C. Ellen van der Schoot MD, PhD^9^, Tineke C.T.M. van der Pouw Kraan PhD^7^, Anton J. Horrevoets PhD^7^, Anja M. van der Laan MD, PhD^1^, Hans W.M. Niessen MD, PhD^4,6,8^, Jan J. Piek MD, PhD^1,8^

Amsterdam UMC, University of Amsterdam, Department of ^1^Cardiology, ^2^Biomedical Engineering and Physics, , , Meibergdreef 9, Amsterdam, The Netherlands;

^3^Netherlands Heart Institute, Moreelsepark 1, Utrecht, The Netherlands;

Amsterdam UMC, VU University Amsterdam, Departments of ^4^Pathology, ^5^Cardiology, ^6^Cardiac Surgery, ^7^Molecular Cell Biology and Immunology, de Boelelaan 1117, Amsterdam, The Netherlands;

^8^Amsterdam Cardiovascular Sciences, Amsterdam, The Netherlands;

^9^Department of Experimental Immunohematology, Sanquin Research, , Plesmanlaan 125, Amsterdam, The Netherlands;

^10^Radboud University Medical Centre, Department of Cardiology, Geert Grooteplein Zuid 10, Nijmegen, The Netherlands

Erasmus Medical Centre, departments of ^11^Cardiology, ^12^Cardiology and Radiology, Dr. Molewaterplein 40 , Rotterdam, The Netherlands.

*corresponding author: de Boelelaan 1117, 1081HV, Amsterdam, Tel: +31 20 4444356, Fax: +31 20 696 2609, e-mail: e.terhorst@vumc.nl

# Supplementary methods

## Patient CMR imaging and data analysis

Patients were studied on a 1.5 (*n* = 50) or 3.0 (*n* = 1) Tesla clinical scanner. CMR acquisition and analyses were performed according to a standardized protocol which has been previously described.^1, 2^ In short, contiguous short axis cine images were acquired every 10 mm covering the whole left ventricle from base to apex, using a segmented steady-state free precession pulse sequence. Late gadolinium-enhanced (LGE) images were acquired 10 to 15 minutes after administration of a gadolinium-based contrast agent (0.2 mmol/kg Dotarem; Guerbet, Gorinchem, the Netherlands), using a 2D segmented inversion recovery gradient-echo pulse sequence, with slice position identical to the cine images.

Analyses of CMR data were performed in one core laboratory, blinded for data regarding monocyte characteristics, using dedicated software (Mass, Medis, Leiden, the Netherlands). LV volumes were measured on the cine images and indexed for body surface area. LV ejection fraction was calculated. On LGE images, areas of hyperenhancement were defined using the full-width at half-maximum method.^3^

## Human monocyte isolation and whole genome transcriptome analysis

After preparation of the PBMC suspension using density gradient centrifugation,^1, 4^ a small portion of the PBMC cell suspension was immediately sent to the central cell-processing laboratory at Sanquin Research (Amsterdam, The Netherlands) and cryopreserved for further analyses. Monocytes were isolated from the PBMC suspension using CD14 immunomagnetic microbeads (Miltenyi Biotec, Auburn, CA). A subsample was stained with SYTOX Blue Nucleic Acid (ThermoFisher Scientific, Waltham, MA, USA) to identify non-viable false positive CD14+ cells, which was 2,3 ± 2,0 % of the total CD14+ isolated population (Figure S1). The isolated monocytes were lysed, and total RNA was micro-purified using the mirVana PARIS kit according to the manufacturer’s protocol (Ambion, Austin, TX). Subsequently, RNA was amplified and biotinylated using the Illumina TotalPrep RNA Amplification kit (Ambion), and samples were randomly hybridized to HumanHT-12 Expression BeadChips (Illumina, San Diego, CA) at ServiceXS (Leiden, The Netherlands), followed by scanning and feature extraction.

*Statistical analysis for transcriptome analysis*

For transcriptome analysis, bead summary intensities were log2-transformed and quantile normalized using the limma package^5^ and scripts in R/Bioconductor.^6, 7^ Statistical analysis for microarray data (SAM) was used to identify genes that associated with the relative change (decrease or increase in LV EDVi), and infarct size (below and above median) and presence of microvascular obstruction at baseline.^8^ We performed pathway analysis on the relative change in LV EDVi as continuous variable in SAM, using the gene sets from the molecular signatures database provided by the Broad institute, Cambridge, MA (gene set C2 version 3.0, containing 3272 gene sets).^9, 10^ Genes and pathways with a *P*-value < 0.05 and a false discovery rate < 5% were considered significant. RVista was used to analyze transcription factor binding sites of genes that were significantly associated with the relative change in LV EDVi, using the conserved sequences of the human Transfac 2006 database for the analysis of the 500 base pair upstream regulatory regions.^11^

## Verification of human gene array results by real-time RT-PCR

RNA was reversed transcribed into cDNA, using the RevertAid H Minus First Strand cDNA Synthesis Kit (Fermentas, St. Leon-Rot, Germany). The mRNA expression levels of IFIT1, MX2, CXCL10 and STAT1 were measured by real-time reverse transcriptase-polymerase chain reaction using the primer pairs sequences presented in Table S1A. Gene expression levels were corrected for the expression level of GAPDH and displayed as relative expression values.

## Rat echocardiography

Cardiac function was measured using 2D-echocardiograpy at 4 days prior to MI (baseline), 3 days following MI (day 3) and prior to sacrifice (day 28) using a 13 MHz linear-array transducer (ProSound SSD-4000 PureHD, Aloka, Tokyo, Japan). Images were analyzed using Sante DICOM Viewer program (Santesoft LT, Athens, Greece). LV chamber area was measured using the short axis and LV internal diameter of the diastole (LVIDd) and systole (LVIDs) were measured at the maximal and minimal diameters respectively, acquired from the M-mode at the level selected for visualization of the papillary muscle. FS was calculated as the change in LVIDd and LVIDs normalized to LVIDd ((LVIDd-LVIDs)/LVIDd).

## Rat infarct size assessment

At termination of the experiment, rats were killed by excision of the heart under deep anesthesia using 5% isoflurane. The heart was excised and cut into five equal slices.^12^ To discriminate viable myocardium from infarcted myocardium, histochemical staining with phosphotungstic acid hematoxylin was performed as described earlier.^13^ Stained slides were scanned and the infarcted area and total heart area were marked manually using the program Pannoramic viewer (version 1.15.4, 3D Histech Ltd, Budapest, Hungary). The average infarct size of all five slices was calculated as a percentage of the complete transverse heart section.

## Immunohistochemistry

Paraffin embedded slides were deparaffinized, rehydrated and subsequently incubated in methanol/H_2_O_2_ (0.3%) for 30 minutes to block endogenous peroxidases. Macrophages were stained using a mouse-anti-rat CD68 antibody (ED1; 1:400, RT, 60 min; Serotec) following antigen retrieval with 0.1% pepsin (in 0.02 M HCl, 37°C, 30 min). To stain the reparative macrophage subset, the mouse-anti-rat CD163 primary antibody (clone ED2, 1:100, RT, 60 min; kindly provided by prof. C.D. Dijkstra, VUmc, Amsterdam, the Netherlands) was used after antigen retrieval by boiling the slides in 10 mM sodium citrate buffer (pH 6.0) for 10 min. After a wash with PBS, all sections were incubated for 30 minutes at RT with the EnVision-HRP Detection kit (Dako, Copenhagen, Denmark). Staining complexes were visualised using 3,3’-diaminobenzidine (DAB, 0.1 mg/ml, 0.02% H_2_O_2_, Dako) and sections were subsequently counterstained with hematoxylin, dehydrated and covered. Covered sections were scanned digitally and the stained area was subsequently analyzed using ImageJ software (version 1.49v. National Institutes of Health, USA).^14^ Control slides incubated with PBS instead of primary antibody yielded no staining (not shown).

## Rat monocyte subsets flow cytometry

Rat blood was collected in heparin coated tubes using an tail vein incision on 4 days prior to MI or sham (baseline), day 2, 3 and 28. To analyse monocyte subsets (i.e. RP-1^-^/CD172a^+^cells subdivided into the proinflammatory CD43-lo or the reparative CD43-hi subset), full blood was incubated with PE-conjugated mouse anti-rat granulocytes (1:10, clone RP-1, BD Bioscience, Vianen, the Netherlands), FITC-conjugated mouse anti-rat CD172a (1:20, clone MRC OX-41, Cedarlane, Hornby, Ontario, Canada) and AlexaFluor647-conjugated mouse anti-rat CD43 (1:200, clone W3/13, Biolegend, San Diego, CA, USA) for 30 minutes at RT in the dark. Erythrocytes were lysed using lysis buffer (containing 1.70 mM NH4Cl, 0.01 mM KHCO3 and 1.30 µM tetra sodium EDTA in water; pH = 7.3) for 8 minutes. After two washes in 1% bovine serum albumin in PBS at 4ºC, cells were measured using the Gallios flow cytometer (10 colors, Beckman Coulter, Indianapolis, IN, USA). Data was analyzed with Kaluza software (version 1.5, Beckman Coulter). Live cells were firstly selected using the forward/side scatter plot and subsequently selected for RP-1 negativity and CD172a positivity to discriminate the monocytes from the granulocytes. Monocytes were distinguished in CD43-lo and CD43-hi subsets using the CD172a/CD43 dot plot.^15^

## Rat PBMC collection and real time RT-PCR

Rat blood was diluted 1:1 in PBS and PBMC were collected using density gradient centrifugation (Lymphoprep™ Axis-Shield PoC AS, Oslo, Norway) according to the manufacturer’s protocol. PBMC were lysed for five minutes in TRIzol® reagent (Fisher Scientific, Landsmeer, the Netherlands) and stored at -80°C until further analysis. RNA was isolated according to the manufacture’s protocol (TRIzol®, Fisher Scientific) and diluted in water. Concentration and quality of the RNA was measured using the NanoDrop 2000 Spectrophotometer (ThermoFisher Scientific) and reversed transcribed into cDNA using the RevertAid H Minus First Strand cDNA Synthesis Kit. Expression levels of CXCL10 and STAT1 were measured using primer pairs as presented in Table S1B during real-time RT-PCR using fast SYBR™ green master mix (ThermoFisher Scientific).

Supplementary tables

**Table S1A.** Sequences of human oligonucleotide primers used for real time RT-PCR

| **Symbol (human)** |  | **Forward oligonucleotide** | **Reverse oligonucleotide** |
| --- | --- | --- | --- |
| **IFITM1** |  | CCCTCTTCTTGAACTGGTGCTG | CCTGTCCCTAGACTTCACGGAGTA |
| **MX2** |  | CTGTTCAGAGCACGATTGAAGAC | TCTGCCTTTGCTGTGTGTTTC |
| **CXCL10** |  | CGCTGTACCTGCATCAGCAT | CATCTCTTCTCACCCTTCTTTTTCA |
| **STAT1** |  | TGGCACCAGAACGAATGAGG | ACCAGGCTGGCACAATTGG |
| **GAPDH** |  | GCCAGCCGAGCCACATC | TGACCAGGCGCCCAATAC |

**Table S1B.** Sequences of rat oligonucleotide primers used for real time RT-PCR

| **Symbol (rat)** |  | **Forward oligonucleotide** | **Reverse oligonucleotide** |
| --- | --- | --- | --- |
| **CXCL10** |  | GAAAGCGGTGAGCCAAAGAA | CACACTGGGTAAAGGGAGGT |
| **GAPDH** |  | ATGGCCTTCCGTGTTCCTAC | CTGCTTCACCACCTTCTTGATG |

**Table S2** Patient characteristics at baseline

|  | Patients  (*n* = 51) |  | Decreased  LV EDVi  (*n* = 14) | Increased  LV EDVi  (*n* = 37) | *P*-  value |
| --- | --- | --- | --- | --- | --- |
| Age (yr) | 56 ± 9 |  | 58 ± 9 | 56 ± 9 | 0.33 |
| Male gender | 42 (82) |  | 10 (71) | 32 (87) | 0.24 |
| Coronary risk factors |  |  |  |  |  |
| Diabetes mellitus | 5 (10) |  | 1 (7) | 4 (11) | >0.99 |
| Hypertension | 10 (20) |  | 0 (0) | 10 (27) | 0.05 |
| Family history of coronary heart  disease | 23 (45) |  | 4 (29) | 19 (51) | 0.21 |
| Hypercholesterolemia | 11 (22) |  | 4 (29) | 7 (19) | 0.47 |
| Current smoking | 27 (53) |  | 8 (57) | 19 (51) | 0.46 |
| Angiography and infarct treatment |  |  |  |  |  |
| Time from symptom onset to  reperfusion (hrs.) | 3.0 [2.2–4.8] |  | 3.3 [2.4-5.0] | 3.0 [2.2-4.7] | 0.74 |
| Infarct-related artery |  |  |  |  | 0.30 |
| Left anterior descending artery | 34 (67) |  | 7 (50) | 27 (73) |  |
| Left circumflex artery | 5 (10) |  | 2 (14) | 3 (8) |  |
| Right coronary artery | 12 (24) |  | 5 (36) | 7 (19) |  |
| Multivessel disease | 15 (30) |  | 7 (50) | 8 (22) | 0.08 |
| Cardiovascular magnetic resonance imaging |  |  |  |  |  |
| Baseline LV EF (%) | 42 ± 10 |  | 44 ± 8 | 41 ± 10 | 0.32 |
| Baseline LV EDVi (mL/m^2^) | 98 ± 16 |  | 103 ± 15 | 96 ± 16 | 0.18 |
| Baseline LV ESVi (mL/m^2^) | 58 ± 17 |  | 58 ± 15 | 58 ± 17 | 0.91 |
| Baseline infarct size (% of LV) | 18 ± 9 |  | 14 ± 10 | 20 ± 9 | 0.08 |
| Cell characteristics |  |  |  |  |  |
| Monocytes (*10^6^/L) | 726 ± 215 |  | 694 ± 182 | 739 ± 227 | 0.51 |

Values are expressed as number (%), mean ± SD, or median [25th–75th percentile]. LV EF denotes left ventricular ejection fraction; LV EDVi, left ventricular end-diastolic volume index; LV ESVi, left ventricular end-systolic volume index.

**Table S3** List of differentially expressed genes

| Symbol | Gene | Score | Fold  change | q-value (%) |
| --- | --- | --- | --- | --- |
| AGRN | agrin | -3,83 | 0,84 | 0,00 |
| HEG1 | HEG homolog 1 | -3,66 | 0,85 | 0,00 |
| SAMD9L | sterile alpha motif domain containing 9-like | -3,62 | 0,62 | 0,00 |
| IFIT2 | interferon-induced protein with tetratricopeptide repeats 2 | -3,59 | 0,52 | 0,00 |
| LBA1 | lupus brain antigen 1 | -3,50 | 0,82 | 0,00 |
| SERPING1 | serpin peptidase inhibitor, clade G (C1 inhibitor), member 1 | -3,47 | 0,62 | 0,00 |
| GBP4 | guanylate binding protein 4 | -3,45 | 0,62 | 0,00 |
| CXCL10 | chemokine (C-X-C motif) ligand 10 | -3,39 | 0,61 | 0,00 |
| IFIT1 | interferon-induced protein with tetratricopeptide repeats 1 | -3,33 | 0,49 | 0,00 |
| SEPT4 | septin 4 | -3,33 | 0,81 | 0,00 |
| MX2 | myxovirus (influenza virus) resistance 2 | -3,32 | 0,64 | 0,00 |
| FTSJD2 | FtsJ methyltransferase domain containing 2 | -3,28 | 0,83 | 0,00 |
| PRIC285 | peroxisomal proliferator-activated receptor A interacting complex 285 | -3,28 | 0,67 | 0,00 |
| DHX58 | DEXH (Asp-Glu-X-His) box polypeptide 58 | -3,23 | 0,75 | 0,00 |
| PARP9 | poly (ADP-ribose) polymerase family, member 9 | -3,21 | 0,67 | 0,00 |
| PARP14 | poly (ADP-ribose) polymerase family, member 14 | -3,19 | 0,74 | 0,00 |
| GIMAP5 | GTPase, IMAP family member 5 | -3,16 | 0,71 | 4,88 |
| IFI35 | interferon-induced protein 35 | -3,15 | 0,71 | 4,88 |
| STAT1 | signal transducer and activator of transcription 1 | -3,14 | 0,63 | 4,88 |
| HERC5 | hect domain and RLD 5 | -3,14 | 0,57 | 4,88 |
| IFIT3 | interferon-induced protein with tetratricopeptide repeats 3 | -3,10 | 0,53 | 4,88 |
| LOC55908 | hepatocellular carcinoma-associated gene TD26 | -3,07 | 0,90 | 4,88 |
| XAF1 | XIAP associated factor 1 | -3,06 | 0,61 | 4,88 |
| TAP1 | transporter 1, ATP-binding cassette, sub-family B | -3,04 | 0,70 | 4,88 |
| HSPA6 | heat shock 70kDa protein 6 (HSP70B') (HSPA6), mRNA. | -3,02 | 0,72 | 4,88 |
| CCL8 | chemokine (C-C motif) ligand 8 | -3,01 | 0,78 | 4,88 |
| TAP2 | transporter 2, ATP-binding cassette, sub-family B | -3,00 | 0,80 | 4,88 |
| RARRES3 | retinoic acid receptor responder (tazarotene induced) 3 | -3,00 | 0,76 | 4,88 |
| SAMD9 | sterile alpha motif domain containing 9 | -3,00 | 0,72 | 4,88 |
| GMPR | guanosine monophosphate reductase | -2,99 | 0,83 | 4,88 |
| BST2 | bone marrow stromal cell antigen 2 | -2,99 | 0,81 | 4,88 |
| RSAD2 | radical S-adenosyl methionine domain containing 2 | -2,99 | 0,70 | 4,88 |
| WARS | tryptophanyl-tRNA synthetase | -2,98 | 0,75 | 4,88 |
| GBP2 | guanylate binding protein 2, interferon-inducible | -2,97 | 0,75 | 4,88 |
| PARP3 | poly (ADP-ribose) polymerase family, member 3 | -2,96 | 0,86 | 4,88 |
| C2 | complement component 2 | -2,96 | 0,83 | 4,88 |
| STAT1 | signal transducer and activator of transcription 1 | -2,95 | 0,66 | 4,88 |
| STAT1 | signal transducer and activator of transcription 1 | -2,94 | 0,66 | 4,88 |
| OAS1 | 2',5'-oligoadenylate synthetase 1, 40/46kDa | -2,94 | 0,66 | 4,88 |
| ADAR | adenosine deaminase, RNA-specific | -2,92 | 0,81 | 4,88 |
| BTN3A1 | butyrophilin, subfamily 3, member A1 | -2,92 | 0,80 | 4,88 |
| HERC6 | hect domain and RLD 6 | -2,91 | 0,79 | 4,88 |
| ANKRD22 | ankyrin repeat domain 22 | -2,90 | 0,73 | 4,88 |
| C1QC | complement component 1, q subcomponent, C chain | -2,89 | 0,75 | 4,88 |
| UBE2L6 | ubiquitin-conjugating enzyme E2L 6 | -2,88 | 0,74 | 4,88 |
| NR1H3 | nuclear receptor subfamily 1, group H, member 3 | -2,87 | 0,80 | 4,88 |

List of genes that are differentially expressed between patients with an increased and patients with a decreased LV EDVi at 4 months after AMI, analyzed by (Sam) ref: Tusher PNAS 2001. All genes were lower expressed in the patients with an increased LV EDVi.

**Table S4** Verification of gene array results with real-time RT-PCR

|  |  |  | **Gene array** | |  | **RT-PCR** | |
| --- | --- | --- | --- | --- | --- | --- | --- |
| **Symbol** | **Gene** |  | **Ratio** | **FDR** |  | **Ratio** | ***P*-value** |
| CXCL10 | chemokine (C-X-C motif) ligand 10 |  | 0.61 | 0.00 |  | 0.42 | 0.001 |
| MX2 | myxovirus (influenza virus) resistance 2 |  | 0.64 | 0.00 |  | 0.49 | 0.02 |
| IFIT1 | interferon-induced protein with  tetratricopeptide repeats 1 |  | 0.49 | 0.00 |  | 0.31 | 0.007 |
| STAT1 | signal transducer and activator of  transcription 1 |  | 0.66 | 4.88 |  | 0.60 | 0.006 |

Lower expression of IFN response genes in patients with an increased LV EDVi at 4 months follow-up was confirmed using RT-PCR. FDR denotes false discovery rate.

**Table S5** Transcription factor binding site analysis

| **Symbol** | **Gene** | **Number of hits in**  **submitted regions** | **Total number of hits on genome** | ***P*-value** |
| --- | --- | --- | --- | --- |
| ICSBP | interferon consensus sequence-  binding protein | 9 | 388 | 1.79*10^-9^ |
| IRF-1 | Interferon regulatory factor 1 | 23 | 4429 | 4.77*10^-8^ |
| ISGF-3 | Interferon-stimulated gene factor 3 | 4 | 133 | 8.11*10^-7^ |
| BLIMP1 | B lymphocyte-induced maturation  protein 1 | 6 | 413 | 8.21*10^-7^ |

Genes that were expressed at significantly lower levels (FDR < 5%) in AMI patients with an increased LV EDVi at 4 months follow-up were analyzed for the presence of overrepresented transcription factor binding sites in their promoter regions in comparison to the frequency of these sites in the whole genome. The top 4 significant transcription factors are all related to IFN signaling (sorted by *P*-value).

***Cxcl10***

**Supplementary figure S1**

1

**Supplementary Figure S2**

**
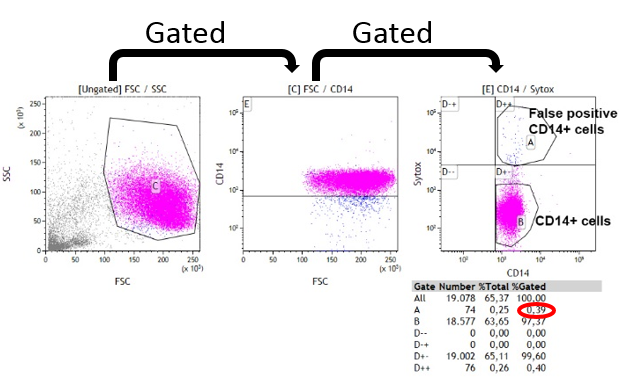
**

**Supplementary figure legends**

**Figure S1 IFN-α administration in experimental MI alters IFN response gene expression in rat PBMC.** Expression levels at day 2 and day 3 following MI and IFN-α administration in rat PBMCs of *Cxcl10* normalized to GAPDH expression. PBMC = peripheral blood mononuclear cells. IFN-α = Interferon alpha. Differences in results are tested using a Mann-Whitney U test (day 2) or an Wilcoxon signed rank test (MI day 2 to day 3).

**Figure S2. Gating strategy to identify non-viable CD14^+^ cells.** After CD14+ Macs beads isolation, a subsample was stained with SYTOX to stain non-viable CD14^+^ cells. Primarily, cells were gated based upon their size and granularity using a FSC-SSC dot blot (most left). Subsequently, the CD14^+^ positive cells were gated. From this, CD14^+^SYTOX^-^ and CD14^+^SYTOX^+^ cells were gated to differentiate between the viable and non-viable CD14^+^ cells. The percentage false positive CD14^+^ cells in this sample is highlighted with a red circle.

# References

1. Hirsch A, Nijveldt R, van der Vleuten PA, Biemond BJ, Doevendans PA, van Rossum AC, Tijssen JG, Zijlstra F, Piek JJ, investigators H. Intracoronary infusion of autologous mononuclear bone marrow cells or peripheral mononuclear blood cells after primary percutaneous coronary intervention: Rationale and design of the hebe trial--a prospective, multicenter, randomized trial. *Am Heart J*. 2006;152:434-441

2. Hirsch A, Nijveldt R, van der Vleuten PA, Tio RA, van der Giessen WJ, Marques KM, Doevendans PA, Waltenberger J, Ten Berg JM, Aengevaeren WR, Biemond BJ, Tijssen JG, van Rossum AC, Piek JJ, Zijlstra F. Intracoronary infusion of autologous mononuclear bone marrow cells in patients with acute myocardial infarction treated with primary pci: Pilot study of the multicenter hebe trial. *Catheter Cardiovasc Interv*. 2008;71:273-281

3. Flett AS, Hasleton J, Cook C, Hausenloy D, Quarta G, Ariti C, Muthurangu V, Moon JC. Evaluation of techniques for the quantification of myocardial scar of differing etiology using cardiac magnetic resonance. *Jacc-Cardiovasc Imag*. 2011;4:150-156

4. van Beem RT, Hirsch A, Lommerse IM, Zwaginga JJ, Noort WA, Biemond BJ, Piek JJ, van der Schoot CE, Voermans C. Recovery and functional activity of mononuclear bone marrow and peripheral blood cells after different cell isolation protocols used in clinical trials for cell therapy after acute myocardial infarction. *EuroIntervention*. 2008;4:133-138

5. Smyth GK. Limma: Linear models for microarray data. In: Gentleman RC, Carey VJ, Dudoit S, Irizarry R, Huber W, eds. *Bioinformatics and computational biology solutions using r and bioconductor*. New York: Springer; 2005:397-420.

6. Gentleman RC, Carey VJ, Bates DM, Bolstad B, Dettling M, Dudoit S, Ellis B, Gautier L, Ge Y, Gentry J, Hornik K, Hothorn T, Huber W, Iacus S, Irizarry R, Leisch F, Li C, Maechler M, Rossini AJ, Sawitzki G, Smith C, Smyth G, Tierney L, Yang JY, Zhang J. Bioconductor: Open software development for computational biology and bioinformatics. *Genome Biol*. 2004;5:R80

7. Rainer J, Sanchez-Cabo F, Stocker G, Sturn A, Trajanoski Z. Carmaweb: Comprehensive r- and bioconductor-based web service for microarray data analysis. *Nucleic Acids Res*. 2006;34:W498-503

8. Tusher VG, Tibshirani R, Chu G. Significance analysis of microarrays applied to the ionizing radiation response. *Proc Natl Acad Sci U S A*. 2001;98:5116-5121

9. Mootha VK, Lindgren CM, Eriksson KF, Subramanian A, Sihag S, Lehar J, Puigserver P, Carlsson E, Ridderstrale M, Laurila E, Houstis N, Daly MJ, Patterson N, Mesirov JP, Golub TR, Tamayo P, Spiegelman B, Lander ES, Hirschhorn JN, Altshuler D, Groop LC. Pgc-1alpha-responsive genes involved in oxidative phosphorylation are coordinately downregulated in human diabetes. *Nat Genet*. 2003;34:267-273

10. Subramanian A, Tamayo P, Mootha VK, Mukherjee S, Ebert BL, Gillette MA, Paulovich A, Pomeroy SL, Golub TR, Lander ES, Mesirov JP. Gene set enrichment analysis: A knowledge-based approach for interpreting genome-wide expression profiles. *Proc Natl Acad Sci U S A*. 2005;102:15545-15550

11. Zambon AC, Zhang L, Minovitsky S, Kanter JR, Prabhakar S, Salomonis N, Vranizan K, Dubchak I, Conklin BR, Insel PA. Gene expression patterns define key transcriptional events in cell-cycle regulation by camp and protein kinase a. *Proc Natl Acad Sci U S A*. 2005;102:8561-8566

12. ter Horst EN, Krijnen PAJ, Flecknell P, Meyer KW, Kramer K, van der Laan AM, Piek JJ, Niessen HWM. Sufentanil-medetomidine anaesthesia compared with fentanyl/fluanisone-midazolam is associated with fewer ventricular arrhythmias and death during experimental myocardial infarction in rats and limits infarct size following reperfusion. *Lab Anim*. 2017

13. van Dijk A, Krijnen PA, Vermond RA, Pronk A, Spreeuwenberg M, Visser FC, Berney R, Paulus WJ, Hack CE, van Milligen FJ, Niessen HW. Inhibition of type 2a secretory phospholipase a2 reduces death of cardiomyocytes in acute myocardial infarction. *Apoptosis*. 2009;14:753-763

14. Schneider CA, Rasband WS, Eliceiri KW. Nih image to imagej: 25 years of image analysis. *Nat Methods*. 2012;9:671-675

15. Ahuja V, Miller SE, Howell DN. Identification of two subpopulations of rat monocytes expressing disparate molecular forms and quantities of cd43. *Cell Immunol*. 1995;163:59-69
